# The roles of behavioral inhibition/activation systems and impulsivity in problematic smartphone use: A network analysis

**DOI:** 10.3389/fpubh.2022.1014548

**Published:** 2022-10-19

**Authors:** Zhihua Guo, Yang He, Tianqi Yang, Lei Ren, Rui Qiu, Xia Zhu, Shengjun Wu

**Affiliations:** Department of Military Medical Psychology, Air Force Medical University, Xi'an, China

**Keywords:** problematic smartphone use, behavioral inhibition system, behavioral activation system, impulsivity, network analysis

## Abstract

**Background:**

Behavioral inhibition/activation systems (BIS/BAS) and impulsivity are associated with problematic smartphone use (PSU). However, no studies to date have explored how the subdomains of BIS/BAS and the dimensions of impulsivity relate to the components of PSU in a joint framework. This study aimed to examine the relationships between the three constructs at a fine-grained level and identify the central nodes and bridge nodes of their relationships using network analysis.

**Methods:**

A regularized partial correlation network of PSU, BIS/BAS, and impulsivity communities was estimated to investigate the connections between variables and determine the expected influence and bridge expected influence for each variable based on data from 325 Chinese adults. PSU, BIS/BAS, and impulsivity were assessed using the Smartphone Application-Based Addiction Scale (SABAS), BIS/BAS scales, and Barratt Impulsiveness Scale-Version 11 (BIS-11), respectively.

**Results:**

In addition to connections within each community, network analysis revealed that there were connections between different communities, especially connections to PSU. I2 “motor impulsivity” was strongly associated with PSU2 “conflict” and PSU6 “relapse”; BASR “BAS-reward responsiveness” was strongly associated with PSU5 “withdrawal.” Nodes BASR “BAS-reward responsiveness” and PSU6 “relapse” were the most central variables, while nodes BASR “BAS-reward responsiveness” and I2 “motor impulsivity” were the strongest bridge variables.

**Conclusion:**

The connections between the subdomains of BIS/BAS and the components of PSU and between the dimensions of impulsivity and the components of PSU may be particularly important in the development and maintenance of PSU. The central variables identified here, along with the bridge variables, could be promising and effective targets for the prevention and intervention of PSU.

## Introduction

The number of smartphone users has surged in recent years because smartphones offer a wide range of functions such as communication, online education, and entertainment. Taking the Chinese population as an example, the China Internet Network Information Center (CNNIC) reported that 1,029 million Chinese people used mobile phones to access the Internet as of December 2021, accounting for 99.7% of Chinese netizens (1). According to a report from Pew Research Center, in 2018, 96% of adults owned a smartphone in South Korea, which was the highest rate of smartphone ownership among the 39 countries surveyed (2). However, with the rapid growth of smartphone users, a related issue—problematic smartphone use (PSU)—has ensued. PSU refers to the compulsive and dependent use of smartphones, leading to negative consequences or impaired daily function (3, 4). The PSU has become a public concern. It has been reported that the median prevalence of PSU amongst children and young people was 23.3% (5). Following suggestions by previous researchers, the current study does not discriminate between PSU and other terms used to describe excessive smartphone use such as “smartphone addiction”; these terms are here collectively referred to as PSU (6, 7). Cumulative evidence indicates that PSU can be related to physical health problems (e.g., headaches, neck and thumb pain) (8–10) and psychological problems (e.g., anxiety and depression, sleep disorders, loneliness, and impulsivity) (11–15). Given the high prevalence and adverse results related to PSU, it is of great significance to reveal the pathogenesis of PSU. According to the Interaction of Person-Affect-Cognition-Execution (I-PACE) model, individuals' predisposing variables (e.g., specific personality trait, neurobiological characteristics, reduced executive functioning and inhibition control, etc.) make them more susceptible to developing PSU (16, 17). Importantly, two candidate transdiagnostic risk factors for PSU are behavioral inhibition/activation systems and impulsivity.

Gray's reinforcement sensitivity theory (RST) has often been used to explain an individual's predisposition to addictive behaviors (18–21). RST attempts to illuminate individual differences in personality traits from the perspective of neurophysiological mechanisms. It holds that there are two brain systems that are sensitive to punishment and rewards, respectively, and control individual behavior: the Behavioral Inhibition System (BIS) and the Behavioral Activation System (BAS) (18, 22, 23). The BIS regulates aversive motivation and is activated by punishment and by the termination of rewards, inhibiting behavior that may result in negative consequences; the BAS regulates appetitive motivation and is activated by rewards and by the termination of punishment, increasing approach behavior and generating positive emotional feelings (20, 22, 24, 25). Growing evidence indicates that BIS/BAS can be associated with addiction or addiction-like behavior, such as substance use disorder (26, 27), Internet addiction (28, 29), and Internet gaming disorder (IGD) (20, 30). Additionally, it has been reported in a prospective study that there are bidirectional interactions between addiction and BIS/BAS (31). Importantly for this study, although few related studies have been conducted, some studies have revealed that BIS/BAS is closely related to PSU and can be risk factors for PSU (25, 32–34). For example, a study has found that BIS is significantly associated with PSU through copula regression analysis (33). Overall, existing researches establish the close relationships between BIS/BAS and PSU.

Impulsivity is a multi-dimensional construct involving actions carried out quickly and without foresight, preferences for risk taking, failures of inhibitory processes, a lack of planning, and a tendency to accept small immediate rewards rather than large delayed rewards and that often results in undesirable consequences (35–39). Impulsive behavior is not always maladaptive: for example, functional impulsivity helps to complete tasks in a limited period of time, and can lead to good outcomes (36, 40). However, excessive and persistent impulsivity is a prominent risk factor for addiction (36, 40–42). It has also been reported that there is a bidirectional relationship between impulsivity and addictive behavior (43). Impulsivity is not only an important predictor of substance use disorders (36, 44, 45) but also a vulnerability factor for non-substance-related addictive disorders (40, 42, 46). Critical for this study, prior studies have revealed that impulsivity is one of the most predictive factors of PSU (3, 25, 40, 41, 47–49). For example, it has been reported that dysfunctional impulsivity is directly connected to PSU, and also indirectly connected to PSU through the mediation of sensation seeking (e.g., thrill and adventure seeking) through stepwise regression (40). Hence, impulsivity has tight relationships with PSU; in particular, it is important for developing, reinforcing, and maintaining the symptoms of PSU. Furthermore, a close relationship between the BAS of the BIS/BAS and impulsivity has been shown in substance use disorders (50, 51).

Prior studies have investigated the relationships between PSU and the BIS/BAS (25) and between PSU and impulsivity based on total scores on scales (41, 47, 49). However, this practice may obscure the variation between individual psychopathological variables and fail to reveal the relationships between the variables at a fine-grained level (52, 53). In fact, of PSU, BIS/BAS, and impulsivity, none are simple unidimensional constructs. PSU consists of six components according to the addiction components model, namely salience (preoccupation with the behavior), mood modification (mood changes brought about by the behavior), tolerance (increasing engagement in the behavior over time), withdrawal (negative feelings and physical symptoms when the behavior is blocked), conflict (interpersonal and intrapersonal relationship problems because of the behavior), and relapse (reversion to the behavior after a period of abstinence) (54, 55). As for BIS/BAS, in addition to BIS, BAS alone is comprised of three subdomains, including reward responsiveness (receptivity to actual or potential rewards), drive (persistent pursuit of goals), and fun seeking (desire for new and potentially rewarding experiences) (18, 22). Impulsivity is composed of three dimensions, including inattention (not focusing on the task at hand), motor impulsiveness (acting on the spur of the moment), and lack of planning (not planning and thinking carefully) (35, 56). Therefore, the single summative score of BIS/BAS, impulsivity, or PSU is based on the notion of variable equivalence and masks the heterogeneity between individual variables, as well as obscure the specific relationships between variables (57, 58). In order to better understand the psychopathology behind PSU, BIS/BAS, and impulsivity at a fine-grained level (especially between PSU and BIS/BAS and between PSU and impulsivity) and to pinpoint effective intervention and prevention targets for PSU, studies are warranted to investigate the relationships among the individual variables of these constructs.

Network analysis is a new and promising data-driven approach that can satisfy this requirement. It involves estimating and visualizing the complex relationships and characteristics of a system in network form (24, 52, 59). The network can consist of individual disorder symptoms (52, 59) or non-symptom factors that may contribute to the development and maintenance of a disorder, such as psychophysiological variables, cognitive process, behaviors, or different personality traits (60, 61). From the perspective of the network theoretical model, psychological constructs are characterized as networks emerging from the interactions between different variables (60, 62). Variables, whether symptoms or non-symptoms, are regarded as the nodes in the psychopathology network; the partial correlations between different variables are represented as node-to-node edges (62, 63).

Network analysis provides further understanding of the mechanisms underlying the development and maintenance of disorders (62, 64). This approach also makes it possible to identify central nodes that greatly affect other nodes across the whole network, as well as bridge nodes, which connect with nodes of other network communities (63–65). The term “community” is used to represent a group of nodes corresponding to a specific construct based on psychological theory or clinical criteria rather than based on any network analytical methods such as community detection (65, 66). Because central nodes have the greatest influence on the overall network and bridge nodes are critical to maintaining the co-occurrence of mental disorders and facilitating the contagion of one disorder to another, these nodes have been identified as more effective targets for prevention and treatment than other nodes (53, 63–65). Network analysis has been used to investigate three or more co-occurring constructs in a joint framework, including problematic Internet and smartphone use, primary emotional systems, and need satisfaction (67), as well as depressive symptoms, parental stress, and mechanistic variables (68).

However, to the best of our knowledge, no study has yet investigated the relationships between PSU, BIS/BAS, and impulsivity in a joint framework using network analysis. To fill that research gap, we used a network analysis approach to examine the interactions between PSU, BIS/BAS, and impulsivity, especially the relationships between subdomains of BIS/BAS and components of PSU and between dimensions of impulsivity and components of PSU. We constructed a PSU-BIS/BAS-Impulsivity network to explore the links among the three communities. We also calculated the expected influence (EI) and bridge expected influence (BEI) of each of the network's nodes to identify central nodes that maintain the whole network and bridge nodes that contribute to the co-occurrence of these constructs. Based on previous studies that BIS/BAS and impulsivity are vulnerability factors of PSU, we hypothesized that subdomains of BIS/BAS and dimensions of impulsivity both exist connections to components of PSU in addition to edges within each community. We also hypothesized that there exist influential nodes in this network that play important roles in the development and maintenance of PSU. In this study, we aimed to advance our understanding of the psychopathological pathways leading from BIS/BAS and impulsivity to PSU, and to determine effective prevention and therapeutic targets for PSU. Considering that no published studies have investigated the relationships between PSU, BIS/BAS, and impulsivity using network analysis, our work is largely exploratory.

## Methods

### Participants

This study was cross-sectional and conducted in China from 27 April 2022 to 16 May 2022. The current study used an online survey hosted on the Wenjuanxing platform (www.wjx.cn), which has been used successfully in previous studies (24, 69). The first part of the survey included the anonymity statement and informed consent form. Participants all gave their informed consent and were allowed to exit the study at any time; they were also encouraged to give honest responses to the survey. A total of 343 participants were recruited through convenience sampling based on WeChat. The inclusion criteria were: (1) healthy adults (aged 18 years or above); (2) college students (undergraduates, masters, or doctors); (3) consent to participate in the study. The exclusion criteria were: (1) a history of organic brain damage or mental disorders; (2) the time used to complete the survey was <100 s, indicating indiscriminate response without consideration. The final sample consisted of 325 participants. The study was reviewed and approved by the Medical Ethics Committee of Tangdu Hospital of the Fourth Military Medical University.

### Measures

In addition to demographic variables such as age, gender, and education, participants were asked to complete scales in the online survey assessing PSU, behavioral inhibition/activation systems (BIS/BAS), and impulsivity.

#### Problematic smartphone use

PSU was measured using the Chinese version of the Smartphone Application-Based Addiction Scale (SABAS) (70–72). The scale consists of 6 items, with each scored from 1 = *strongly disagree* to 6 = *strongly agree*; a higher score indicates a higher level of severity of PSU. The 6 items correspond one-to-one to the 6 core criteria of the components model of addiction, namely salience, conflict, mood modification, tolerance, withdrawal, and relapse (54, 55). The Cronbach's α coefficient for this scale was 0.83 in our sample.

#### Behavioral inhibition/activation systems

BIS/BAS was measured using the Chinese version of the BIS/BAS scales (22, 73). The scale has 18 items in total (items 1 and 18 deleted) that are rated on a 4-point Likert-type scale ranging from 1 = *strongly agree* to 4 = *strongly disagree* (32, 51, 73, 74). The scale includes one subscale assessing BIS (5 items) and 3 subscales assessing BAS (13 items): reward responsiveness (BASR, 4 items), drive (BASD, 4 items), and fun seeking (BASF, 5 items). The Cronbach's α coefficient was 0.82 for BIS, 0.87 for BAS, 0.78 for BASR, 0.75 for BASD, and 0.65 for BASF in the current study.

#### Impulsivity

The valid Chinese version of the Barratt Impulsiveness Scale-Version 11 (BIS-11) was used to assess impulsivity (56, 75). This scale has been widely used in previous studies (76–78). The scale consists of 30 items and can be separated into three dimensions (each with 10 items), namely motor impulsivity, attentional impulsivity, and non-planning impulsivity. Each item is scored from 1 = *never* to 5 = *always*; however, the items of the non-planning and attentional impulsivity dimensions are inversely scored (75). The scores for each dimension and the BIS-11 all range from 0 to 100 after being converted (75). The higher the score, the higher the impulsivity. The internal consistency of the BIS-11 in this study was fairly good; the Cronbach's α coefficients of the motor impulsivity dimension, attentional impulsivity dimension, and non-planning impulsivity dimension were 0.86, 0.89, and 0.83, respectively.

### Analytical procedure

SPSS 26.0 and RStudio software (version 4.1.1) were used to analyze the data. SPSS 26.0 was first used to calculate descriptive metrics and Cronbach's α coefficients. Following previous studies (53, 79, 80), the informativeness of each variable was estimated *via* mean of standard deviation. RStudio was then used to perform network analysis.

We estimated the structure of the networks in the current study *via* Gaussian graphical model (GGM) (81), namely the network structure of PSU components, BIS/BAS subdomains, and impulsivity dimensions (PSU-BIS/BAS-Impulsivity network). Within a GGM, edges are undirected and represent the partial correlation between two nodes after controlling for all remaining nodes. As recommended by a previous study (82), the estimation of network structure was based on Spearman correlations. Moreover, in order to regularize the GGM, we employed the graphical least absolute shrinkage and selection operator (LASSO) method (83). By shrinking all edges and punishing the edges of trivially small partial correlation coefficients to zero, this regularization process helps to remove spurious edges and to obtain a more stable, sparse, and easy-to-interpret network (82, 83). Furthermore, the hyperparameter of the Extended Bayesian Information Criterion (EBIC) was set to 0.5 to determine the optimal network model (82, 84, 85). The network layout was visualized using the Fruchterman-Reingold algorithm (86). In this part of the analytical procedure, we constructed and visualized the network structure using R-package *qgraph* (87).

Both the traditional centrality and bridge centrality measures were reported as raw values. We calculated EI, which was used in this study as the traditional centrality index, using R-package *qgraph* (87). EI is a more suitable than other node centrality measures for evaluating the centrality of each node and determining central nodes in a network with both negative and positive associations (88). EI is defined as the sum of all the edge weights connecting to a given node. A higher EI indicates a more important and influential node in the network. Moreover, to identify bridge nodes in the network that connect different communities or disorders (e.g., PSU, BIS/BAS, and impulsivity), BEI was computed as the bridge centrality indicator using R-package *networktools* (65). BEI refers to the sum of the edge weights connecting a specific node with nodes in the other communities; a higher BEI value represents a higher likelihood of contagion between communities (65). In our study, we defined three communities in the network in advance: we set the six components of PSU to form one community, the four subdomains of BIS/BAS to constitute the second community, and the three impulsivity dimensions to form the third community.

We used R-package *bootnet* to evaluate the robustness and accuracy of the PSU-BIS/BAS-Impulsivity network (86). First, to assess the stability of centrality measures, including node EI and node BEI, we calculated the correlation stability (CS) coefficient using a case-dropping bootstrap approach (1,000 bootstrap samples). The CS coefficient indicates sufficient stability if it is above at least 0.25 (an acceptable value), and preferably >0.5 (86). Second, we examined the accuracy of edge weights with a non-parametric bootstrap method (1,000 bootstrap samples) to compute the 95% confidence interval. A narrower 95% confidence interval indicates a more reliable network (80, 89). Finally, we applied bootstrapped difference tests (1,000 bootstrap samples) to evaluate differences in the centrality indices and edge weights.

## Results

### Descriptive statistics

The mean age of the participants was 21.49 years (SD = 3.73, range = 18–36 years). All participants had received a college education or above. There was an approximately equal gender distribution; 54.8% of the participants were female (*n* = 178), and males made up 45.2% (*n* = 147). The participants reported that they spent an average time of 6.62 h (SD = 3.59) on their smartphones per day. Abbreviations, mean scores, standard deviations, skewness, and kurtosis for each variable of the three constructs are shown in Table 1.

**Table 1:** Abbreviations, mean scores, standard deviations, skewness, and kurtosis for the study variables.

**Variables**	**Abb**	**M**	**SD**	**Skewness**	**Kurtosis**
**Problematic smartphone use**	
Salience	PSU1	3.73	1.31	−0.39	−0.64
Conflict	PSU2	2.77	1.35	0.40	−0.77
Mood modification	PSU3	3.79	1.29	−0.48	−0.45
Tolerance	PSU4	3.43	1.23	0.04	−0.56
Withdrawal	PSU5	3.00	1.29	0.23	−0.60
Relapse	PSU6	3.10	1.22	0.10	−0.65
**Behavioral inhibition/activation systems**	
BIS	BIS	10.03	2.70	0.19	0.18
BAS-reward responsiveness	BASR	6.99	2.18	0.65	0.65
BAS-drive	BASD	8.04	2.07	0.21	0.70
BAS-fun seeking	BASF	10.12	2.28	0.26	0.43
**Impulsivity**	
Non-planning impulsivity	I1	37.54	16.24	0.36	1.07
Motor impulsivity	I2	36.97	14.54	0.07	−0.07
Attentional impulsivity	I3	37.46	12.72	0.66	3.07

Abb, abbreviation; M, mean; SD, standard deviation; PSU, problematic smartphone use; BIS, behavioral inhibition system; BAS, behavioral activation system.

### Network analysis

**Figure 1:**
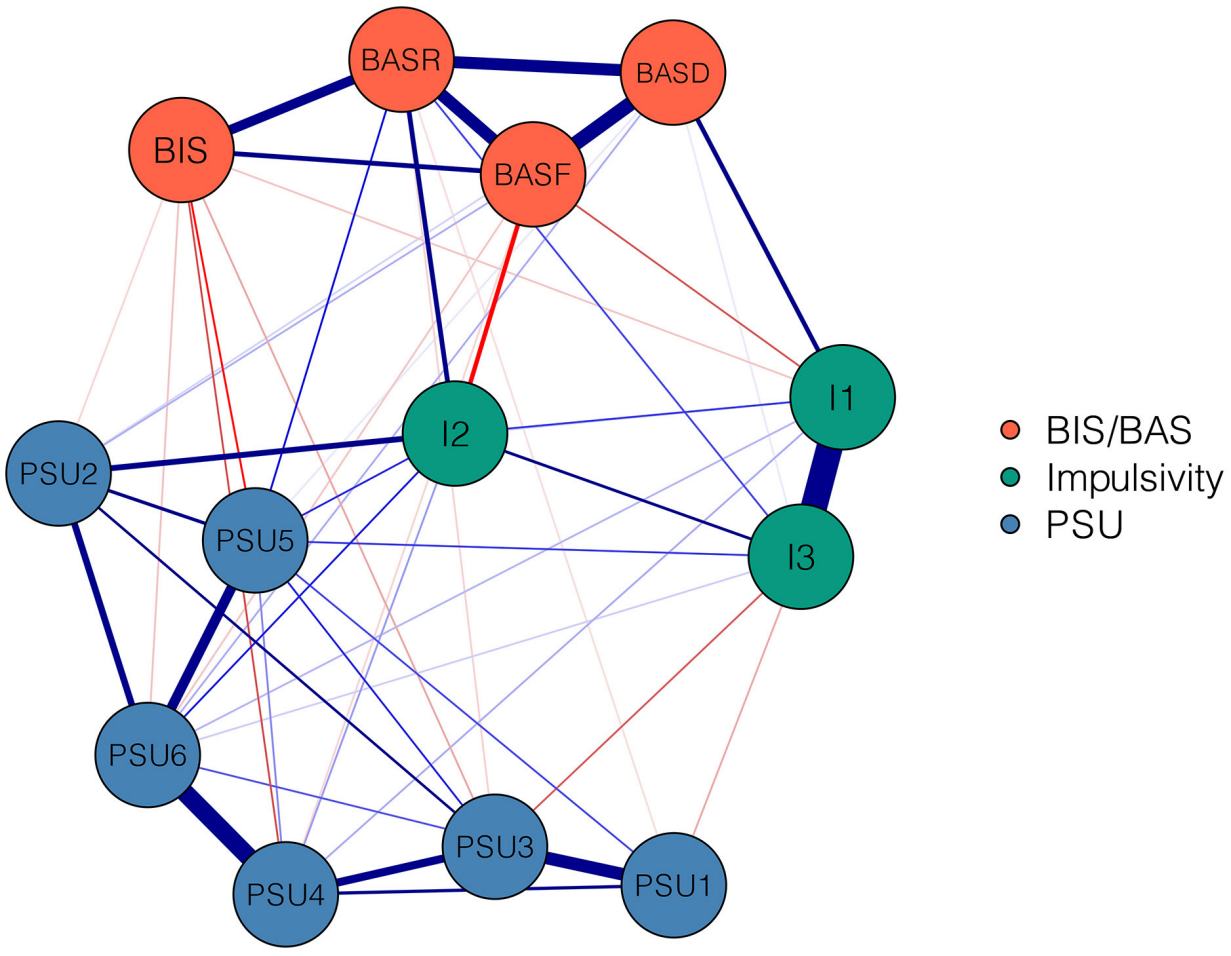
Network Structure of PSU, BIS/BAS, and impulsivity variables. Blue edges represent positive relations, whereas red edges represent negative relations. Thickness of edge indicates strength of relationship. The weights of edges are in Supplementary Table 1. PSU1, salience; PSU2, conflict; PSU3, mood modification; PSU4, tolerance; PSU5, withdrawal; PSU6, relapse; BIS, Behavioral Inhibition System; BASR, Behavioral Activation System-reward responsiveness; BASD, Behavioral Activation System-drive; BASF, Behavioral Activation System-fun seeking; I1, non-planning impulsivity; I2, motor impulsivity; I3, attentional impulsivity.

The level of variable informativeness was checked. No variable was found to be poorly informative, namely 2.5 SD below the mean level of informativeness (M ± SD = 1.28 ± 0.05, 2.31 ± 0.28, and 14.50 ± 1.76 for PSU, BIS/BAS, and impulsivity, respectively). Figure 1 shows the PSU-BIS/BAS-Impulsivity network. In the network with 13 nodes, 52 out of the 78 edges were non-zero, including 14 negative edges and 38 positive edges. The six strongest, positive edges in the final network were identified. Within the PSU community, the two strongest edges were between the nodes PSU1 “salience” and PSU3 “mood modification” (weight = 0.34) and between PSU4 “tolerance” and PSU6 “relapse” (weight = 0.46). Within the BIS/BAS community, the three strongest edges were the edges between the nodes BASD “BAS-drive” and BASF “BAS-fun seeking,” between BASR “BAS-reward responsiveness” and BASF “BAS-fun seeking,” and between BASR “BAS-reward responsiveness” and BASD “BAS-drive” (weight = 0.37, 0.33, 0.32, respectively). Within the impulsivity community, a particularly strong positive edge occurred between nodes I1 “non-planning impulsivity” and I3 “attentional impulsivity” (weight = 0.63). The cross-community edges were weaker than the within-community edges. Nevertheless, some relatively strong edges were identified. I2 “motor impulsivity” was positively associated with PSU2 “conflict” (weight = 0.19) and PSU6 “relapse” (weight = 0.09). BASR “BAS-reward responsiveness” was positively associated with PSU5 “withdrawal” (weight = 0.1). In addition to associations with PSU, some dimensions of impulsivity were positively correlated with subdomains of BIS/BAS. I1 “non-planning impulsivity” was linked to BASD “BAS-drive” (weight = 0.16). I2 “motor impulsivity” was linked to BASR “BAS-reward responsiveness” (weight = 0.16). All edge weights of the PSU-BIS/BAS-Impulsivity network can be seen in Supplementary Table 1. The bootstrapped 95% confidence interval for estimated edge weights is narrow, indicating that the edges were estimated accurately and reliably (see Supplementary Figure 1). The bootstrapped difference test for edge weights is shown in Supplementary Figure 2, revealing that the weights of the six strongest edges were significantly higher than ~84–100% of the weights of other edges.

**Figure 2:**
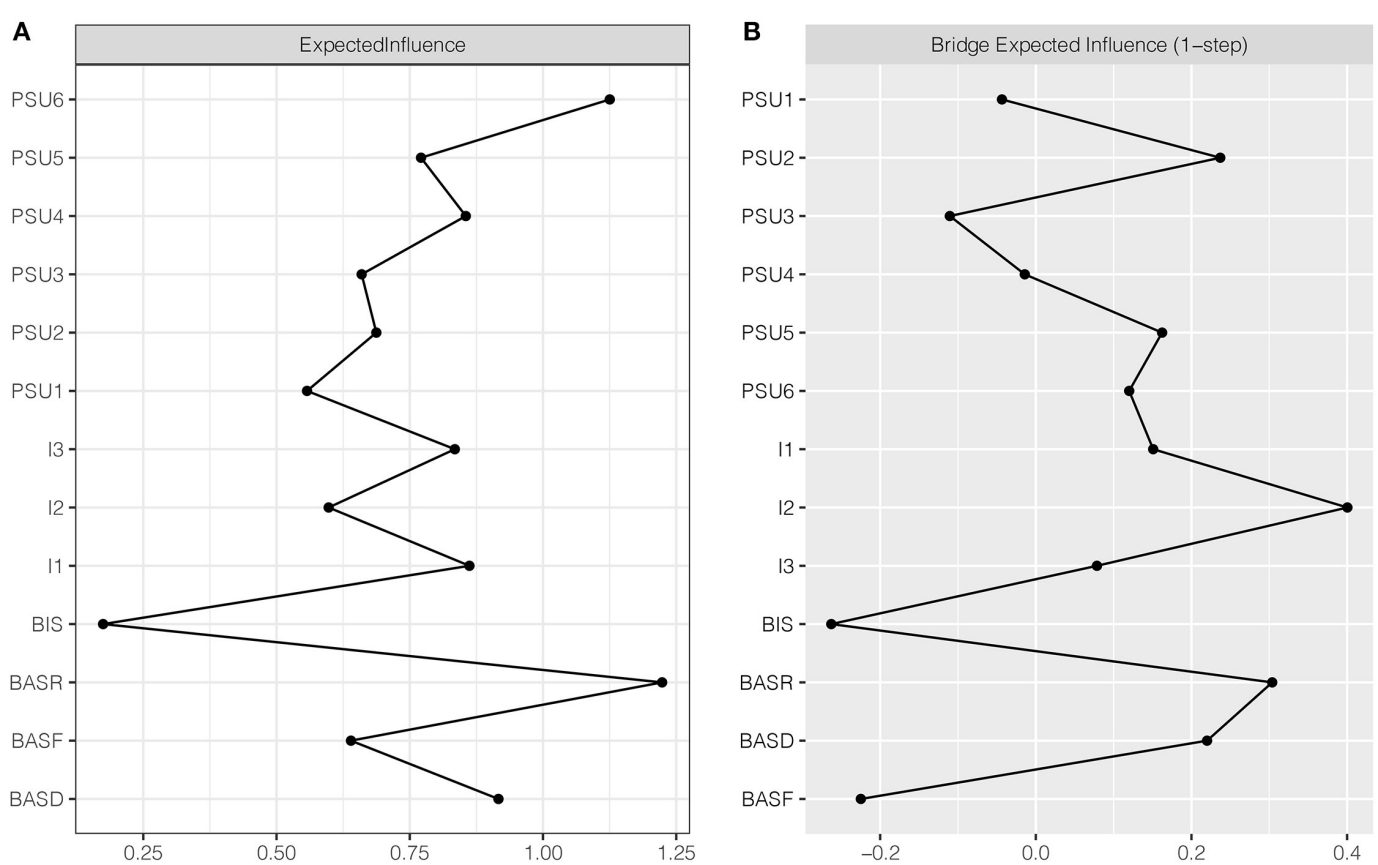
Centrality plot depicting the expected influence and bridge expected influence of each node in the network (raw value). **(A)** Expected influence. **(B)** Bridge expected influence. PSU1, salience; PSU2, conflict; PSU3, mood modification; PSU4, tolerance; PSU5, withdrawal; PSU6, relapse; BIS, Behavioral Inhibition System; BASR, Behavioral Activation System-reward responsiveness; BASD, Behavioral Activation System-drive; BASF, Behavioral Activation System-fun seeking; I1, non-planning impulsivity; I2, motor impulsivity; I3, attentional impulsivity.

The results of the traditional centrality index (i.e., EI) are shown in Figure 2A. The nodes BASR “BAS-reward responsiveness” (EI = 1.22) and PSU6 “relapse” (EI = 1.13) exhibited extremely high EI, marking them as the most central nodes in the network. BIS had the lowest EI and was thus the least central node (EI = 0.17). The CS coefficient for EI was 0.59, exceeding the preferably recommended threshold of 0.5; therefore, the estimation of node EI had a good level of stability (see Supplementary Figure 3). The bootstrapped difference test showed that the EIs of BASR “BAS-reward responsiveness” and PSU6 “relapse” were significantly higher than ~75–92% of the other node EIs; no significant difference was observed in the EIs of these two nodes (see Supplementary Figure 4). To sum up, compared with other nodes, BASR “BAS-reward responsiveness” and PSU6 “relapse” were the most important and influential nodes in the network.

The BEI for each node in the network is shown in Figure 2B. Two bridge nodes exhibited the highest BEI values. One was node BASR “BAS-reward responsiveness” (BEI = 0.3) in the BIS/BAS community; the other was node I2 “motor impulsivity” (BEI = 0.4) in the impulsivity community. The CS coefficient of node BEI was 0.52, indicating that the estimation of BEI was adequately stable (see Supplementary Figure 5). The bootstrapped difference test showed that the BEIs of I2 “motor impulsivity” and BASR “BAS-reward responsiveness” were significantly higher than about 42–67% of the BEIs of the other nodes (see Supplementary Figure 6). Bridge centrality emphasizes the importance of I2 “motor impulsivity” and BASR “BAS-reward responsiveness,” which had enormous influence on the interactions between different communities.

## Discussion

To the best of our knowledge, this is the first study to use network analysis to investigate the relationships between PSU, BIS/BAS, and impulsivity. Based on this approach, this study developed a complex network consisting of three communities: the symptoms of PSU, the subdomains of BIS/BAS, and the dimensions of impulsivity. The strongest edges were observed within each community and there were some relatively weak edges connecting variables between the communities. These results suggest that individual variables of BIS/BAS and impulsivity are linked to specific pathways to develop and maintain PSU. The current study also explored the central nodes and bridge nodes that played important roles in this PSU-BIS/BAS-Impulsivity network. The perspective of network analysis helps to better understand how the dimensions of impulsivity and the subdomains of BIS/BAS are linked to the components of PSU and revealed effective targets for the prevention and treatment of PSU.

This study revealed that the strongest edges appeared within the communities rather than connecting different communities. This is consistent with prior studies that have shown that the strongest edges exist within each community when detecting relationships between variables in a network consisting of two or more communities (24, 53, 63, 90–93). For the PSU community, the two strongest positive edges were between PSU1 “salience” and PSU3 “mood modification” and between PSU4 “tolerance” and PSU6 “relapse”; this has also been found in a prior network research (24). The three strongest edges found within the BIS/BAS community are similar to the results found by other studies using network analysis (94). They were between BASD “BAS-drive” and BASF “BAS-fun seeking,” between BASR “BAS-reward responsiveness” and BASF “BAS-fun seeking,” and between BASR “BAS-reward responsiveness” and BASD “BAS-drive.” For the impulsivity community, the strongest edge was between I1 “non-planning impulsivity” and *I*^3^ “attentional impulsivity,” which is consistent with prior studies that revealed a positive correlation between non-planning impulsivity and attentional impulsivity (95). Additionally, it has been held that non-planning and attentional impulsivity should be regarded as forms of cognitive impulsivity, and thus conceptually different from motor impulsivity (96, 97). This may account for the strong relationship observed between non-planning and attentional impulsivity. Altogether, it is unsurprising that the strongest edges existed within each community rather than connecting different communities, because the variables of each community are sub-components of each psychological construct; these variables have close interactions with each other from a theoretical perspective.

In addition to within-community edges, we found that some variables in the impulsivity and BIS/BAS communities were associated with components of PSU (i.e., cross-community edges), which is consistent with our hypothesis. These findings provide a fine-grained understanding of the links between BIS/BAS and PSU and between impulsivity and PSU. For example, I2 “motor impulsivity” had relatively strong connections with PSU2 “conflict” and PSU6 “relapse,” which could partly account for why individuals with high impulsivity are predisposed to developing PSU (25, 41, 47, 49, 98). It has been reported that response inhibition is closely related to motor impulsivity (99–101). Response inhibition (a component of inhibition control) helps to inhibit impulsive action and to resist temptations, which facilitates adaptive and goal-directed behaviors (102–104). Excessive motor impulsivity may lead to difficulty with response inhibition. Hence, failing to resist temptations may result in relapse and acting impulsively may contribute to conflicts with others (24, 105, 106). Another important edge identified in this study was the positive connection between BASR “BAS-reward responsiveness” and PSU5 “withdrawal,” which is similar to the finding that BASR is a predictor of PSU (25). The current study further explored the possible path linking BAS and PSU, which lies in reward responsiveness and withdrawal. However, this result contradicts a previous study that showed reward responsiveness to be negatively correlated with Internet addiction (20). This inconsistency may arise from a difference in samples: the previous study was based on participants with IGD. IGD can lead to functional and structural alterations of the brain that influence reward responsiveness, contributing to differences from the healthy population (107–110). Since no studies have investigated the relationships between individual variables of BIS/BAS or impulsivity and components of PSU, the current study only provided preliminary insight into an issue certainly worth further study. In addition to edges linking to PSU, there were some edges connecting dimensions of impulsivity and subdomains of BIS/BAS. For example, I1 “non-planning impulsivity” was associated with BASD “BAS-drive,” and I2 “motor impulsivity” was associated with BASR “BAS-reward responsiveness.” These findings are in line with the view that impulsivity is conceptually related to BAS (18, 22, 111, 112). Prior studies based on measuring scales have also reported that BAS is associated with impulsivity (51, 113).

Consistent with our hypothesis that there existed influential nodes (i.e., key central nodes or bridge nodes) in the PSU-BIS/BAS-Impulsivity network, there were two key central nodes with the highest EIs (62, 88): BASR “BAS-reward responsiveness” and PSU6 “relapse.” This result demonstrates that these two variables have the greatest influence within the network and may play the most important role in activating other variables and maintaining the current psychopathological network. This finding aligns with those of previous studies, which have shown that BAS-reward responsiveness exhibits extremely high centrality (24). However, this result differs from prior studies on the centrality indices for components of PSU; relapse was identified as the central node in our study, whereas mood modification was the most central node in an earlier study (24). However, another qualitative examination has revealed that non-addicted smartphone users and addicted users are not different in the components of mood modification and relapse, but are instead different in the other four components of PSU (114). It would seem that mood modification and relapse may not be the dominant components in the development of PSU. Considering that the results of previous studies are inconsistent and confusing, our study is largely exploratory and more studies are warranted.

The BEI is used to evaluate the importance of bridge nodes when analyzing co-occurring constructs (65). Bridge nodes that connect theoretically independent constructs are crucial to understanding the development and maintenance of psychological comorbidities (63, 65). In the current study, I2 “motor impulsivity” and BASR “BAS-reward responsiveness” were identified as key bridge nodes. What interests us most was that I2 “motor impulsivity” had the highest BEI value and was more related to the PSU community than BASR “BAS-reward responsiveness.” This result suggests that motor impulsivity has a significant impact on BAS and the development and maintenance of PSU. These findings echo prior studies that have reported that impulsivity is closely related to PSU (3, 25, 41, 48, 49). Moreover, these findings also accord with some studies that have shown that impulsivity is associated with BAS (51, 113). Our results verify the relationships between impulsivity and PSU from the perspective of network analysis.

These aforementioned findings have important theoretical and clinical implications. Regarding the theoretical implications, this research revealed some edges appeared between BIS/BAS and PSU and between impulsivity and PSU such as edges between I2 “motor impulsivity” and PSU2 “conflict” and between BASR “BAS-reward responsiveness” and PSU5 “withdrawal.” These findings are of importance to figure out specific role played by different components of BIS/BAS or impulsivity in the development and maintenance of symptoms of PSU. In other words, our study shed light on the possible pathological pathways linking BIS/BAS and impulsivity to PSU. Regarding the clinical implications, network theory holds that interventions on critical central nodes may effectively disrupt the overall network and reduce the severity of the entire network, facilitating the intervention and treatment (59, 62, 64, 89). Additionally, targeting key bridge nodes may disrupt the connection between co-occurring constructs and reduce the adverse effects of one disorder on others (i.e., prevent the contagion of one disorder to others), benefiting the treatment outcomes (63–65, 115). In this study, preventing the contagion between communities may decrease the risky interactions between them that may co-lead to PSU. Regarding central nodes, we underscore the importance of taking reward responsiveness and relapse into consideration when attempting to prevent and treat PSU. As for bridge nodes, future intervention of PSU should focus on motor impulsivity and reward responsiveness. Consequently, reward responsiveness of BIS/BAS, relapse of PSU, and motor impulsivity may be effective targets for intervention and treatment of PSU.

The current study has provided preliminary insights into the fine-grained relationships between PSU, BIS/BAS, and impulsivity. Nonetheless, it has some limitations that warrant consideration. First, the results were based on data collected using self-reported scales. Although participants were encouraged to give honest responses, self-reports may nonetheless lead to subjective biases and social approval effects (91, 115). This reminds us to interpret our results cautiously. Second, the study is cross-sectional and cannot verify the causality linking the variables. Future studies should examine the causal relationships using a longitudinal experimental design. Third, similar to the second limitation, although we identified the central and bridge nodes that play important roles in the network and regard them as effective intervention targets (62, 65), it is necessary to verify using further longitudinal or experimental studies whether interventions on these variables will succeed. Fourth, we chose only healthy adults and obtained the network structure and centrality indices, and thus one should be cautious when extending the results to clinical samples. Fifth, similar to fourth limitation, given that this study used a convenient sampling method and enrolled a relatively small sample, the findings are specific to the present sample, and the generalizability of our findings to other populations needs to be established *via* replications in other samples. Sixth, redundant nodes refer to nodes that were highly overlapping and most likely measure the same underlying variable (116). Although it is theoretically justified that none of the variables in our study were redundant (117), it is recommended to conduct statistical redundancy analysis in such future studies. Finally, the network and its characteristics in the current study are specific to the scales we used. We assessed PSU using the SAMAS, BIS/BAS using the BIS/BAS scales, and impulsivity using the BIS-11 scale. This means that the current study did not capture all aspects of these constructs, and only provided a limited picture of the relationships between BIS/BAS or impulsivity and PSU. Future studies can consider other facets of these constructs and integrate them into a unified framework to investigate how BIS/BAS and impulsivity develop and maintain PSU.

## Conclusions

This study is the first to estimate the network structure of PSU, BIS/BAS, and impulsivity based on individual variables and investigate the relationships among these constructs using network analysis. In addition to connections within each community, we found some connections between PSU and BIS/BAS and between PSU and impulsivity. This result showed that there are psychopathological pathways linking BIS/BAS and impulsivity with PSU on a fine-grained level. This study also identified some variables that are critical to the development and maintenance of PSU, including the central nodes BASR “BAS-reward responsiveness” and PSU6 “relapse,” as well as the bridge nodes I2 “motor impulsivity” and BASR “BAS-reward responsiveness”; these findings have important clinical implications, providing promising and effective targets for the prevention and intervention of PSU.

## Data availability statement

The raw data supporting the conclusions of this article will be made available by the authors, without undue reservation.

## Ethics statement

The studies involving human participants were reviewed and approved by the Medical Ethics Committee of Tangdu Hospital of the Fourth Military Medical University. The patients/participants provided their written informed consent to participate in this study.

## Author contributions

ZG, SW, and XZ conceived the study. ZG, YH, and RQ performed the data collection. TY performed data analysis. ZG and YH wrote the draft of the manuscript. LR, SW, and XZ obtained funding and contributed to the manuscript revision. All authors contributed to the article and approved the submitted version.

## Funding

This work was supported by the Key project of PLA Logistics Research Program during the 14th Five-Year Plan period (BKJ21J013).

## Conflict of interest

The authors declare that the research was conducted in the absence of any commercial or financial relationships that could be construed as a potential conflict of interest.

## Publisher's note

All claims expressed in this article are solely those of the authors and do not necessarily represent those of their affiliated organizations, or those of the publisher, the editors and the reviewers. Any product that may be evaluated in this article, or claim that may be made by its manufacturer, is not guaranteed or endorsed by the publisher.
