# Supplementary material for: The roles of behavioral inhibition/activation systems and impulsivity in problematic smartphone use: A network analysis
Source: Front Public Health. 2022 Oct 19;10:1014548. doi: 10.3389/fpubh.2022.1014548 (PMC9627346; doi:10.3389/fpubh.2022.1014548)
Supplement: Supplementary file 1 [file Data_Sheet_1.docx]

Supplementary Materials

1. Table 1. All edges weights within the PSU-BIS/BAS-Impulsivity network

2. Figure 1. Accuracy of edge weights in the PSU-BIS/BAS-Impulsivity network

3. Figure 2. Bootstrapped difference test for edge weights in the PSU-BIS/BAS-Impulsivity network

4. Figure 3. Stability of node expected influences in the PSU-BIS/BAS-Impulsivity network

5. Figure 4. Bootstrapped difference test for node expected influences in the PSU-BIS/BAS-Impulsivity network

6. Figure 5. Stability of node bridge expected influences in the PSU-BIS/BAS-Impulsivity network

7. Figure 6. Bootstrapped difference test for node bridge expected influences in the PSU-BIS/BAS-Impulsivity network

Table 1. All edges weights within the PSU-BIS/BAS-Impulsivity network

|  | PSU1 | PSU2 | PSU3 | PSU4 | PSU5 | PSU6 | I1 | I2 | I3 | BIS | BASR | BASD | BASF |
| --- | --- | --- | --- | --- | --- | --- | --- | --- | --- | --- | --- | --- | --- |
| PSU1 | 0 | 0 | 0.34 | 0.13 | 0.07 | 0.07 | 0 | 0 | -0.03 | 0 | -0.01 | 0 | 0 |
| PSU2 | 0 | 0 | 0.11 | 0 | 0.13 | 0.20 | 0.02 | 0.19 | 0 | -0.01 | 0 | 0.02 | 0.03 |
| PSU3 | 0.34 | 0.11 | 0 | 0.23 | 0.08 | 0 | 0 | 0 | -0.06 | -0.03 | -0.02 | 0 | 0 |
| PSU4 | 0.13 | 0 | 0.23 | 0 | 0.05 | 0.46 | 0.03 | 0.04 | 0 | -0.07 | 0 | 0 | -0.02 |
| PSU5 | 0.07 | 0.13 | 0.08 | 0.05 | 0 | 0.28 | 0 | 0.08 | 0.08 | -0.1 | 0.1 | 0.01 | 0 |
| PSU6 | 0.07 | 0.2 | 0 | 0.46 | 0.28 | 0 | 0.03 | 0.09 | 0.02 | -0.02 | 0 | 0.03 | -0.02 |
| I1 | 0 | 0.02 | 0 | 0.03 | 0 | 0.03 | 0 | 0.08 | 0.63 | -0.02 | 0 | 0.16 | -0.06 |
| I2 | 0 | 0.19 | 0 | 0.04 | 0.08 | 0.09 | 0.08 | 0 | 0.12 | 0 | 0.16 | 0 | -0.16 |
| I3 | -0.03 | 0 | -0.06 | 0 | 0.08 | 0.02 | 0.63 | 0.12 | 0 | 0 | 0.07 | 0.01 | 0 |
| BIS | 0 | -0.01 | -0.03 | -0.07 | -0.1 | -0.02 | -0.02 | 0 | 0 | 0 | 0.27 | 0 | 0.16 |
| BASR | -0.01 | 0 | -0.02 | 0 | 0.1 | 0 | 0 | 0.16 | 0.07 | 0.27 | 0 | 0.32 | 0.33 |
| BASD | 0 | 0.02 | 0 | 0 | 0.01 | 0.03 | 0.16 | 0 | 0.01 | 0 | 0.32 | 0 | 0.37 |
| BASF | 0 | 0.03 | 0 | -0.02 | 0 | -0.02 | -0.06 | -0.16 | 0 | 0.16 | 0.33 | 0.37 | 0 |

Figure 1. Accuracy of edge weights in the PSU-BIS/BAS-Impulsivity network

*Note*: The red line depicts the sample edge weights and the gray bar depicts the bootstrapped confidence interval.

Figure 2. Bootstrapped difference test for edge weights in the PSU-BIS/BAS-Impulsivity network

*Note*: Gray boxes indicate edge weights that do not differ significantly from one another, while black boxes indicate edge weights that do differ significantly. Blue and red boxes on the diagonal correspond to edge weights with positive and negative correlations, respectively.

Figure 3. Stability of node expected influences in the PSU-BIS/BAS-Impulsivity network

*Note*: The red bar represents the average correlation between node expected influences in the full sample and subsample with the red area depicting the 2.5th quantile to the 97.5th quantile.

Figure 4. Bootstrapped difference test for node expected influences in the PSU-BIS/BAS-Impulsivity network

*Note*: Gray boxes indicate node expected influences that do not differ significantly from one another, while black boxes indicate node expected influences that do differ significantly. The numbers in the white boxes (i.e., diagonal line) represent the values of node expected influences.

Figure 5. Stability of node bridge expected influences in the PSU-BIS/BAS-Impulsivity network

*Note*: The red bar represents the average correlation between node bridge expected influences in the full sample and subsample with the red area depicting the 2.5th quantile to the 97.5th quantile.

Figure 6. Bootstrapped difference test for node bridge expected influences in the PSU-BIS/BAS-Impulsivity network

*Note*: Gray boxes indicate node bridge expected influences that do not differ significantly from one another, while black boxes indicate node bridge expected influences that do differ significantly.
